# Supplementary material for: SP1 and STAT3 Functionally Synergize to Induce the RhoU Small GTPase and a Subclass of Non-canonical WNT Responsive Genes Correlating with Poor Prognosis in Breast Cancer
Source: Cancers (Basel). 2019 Jan 16;11(1):101. doi: 10.3390/cancers11010101 (PMC6356433; doi:10.3390/cancers11010101)
Supplement: Supplementary file 1 [file cancers-11-00101-s001.zip › cancers-422538-SI.pdf]

Supplementary Materials: SP1 and STAT3 Functionally Synergize to Induce the RhoU Small GTPase and a Subclass of Non-Canonical WNT Responsive Genes Correlating with Poor Prognosis in Breast Cancer

Emanuele Monteleone, Valeria Orecchia, Paola Corrieri, Davide Schiavone, Lidia Avalle, Enrico Moiso, Aurora Savino, Ivan Molineris, Paolo Provero and **Valeria Poli**

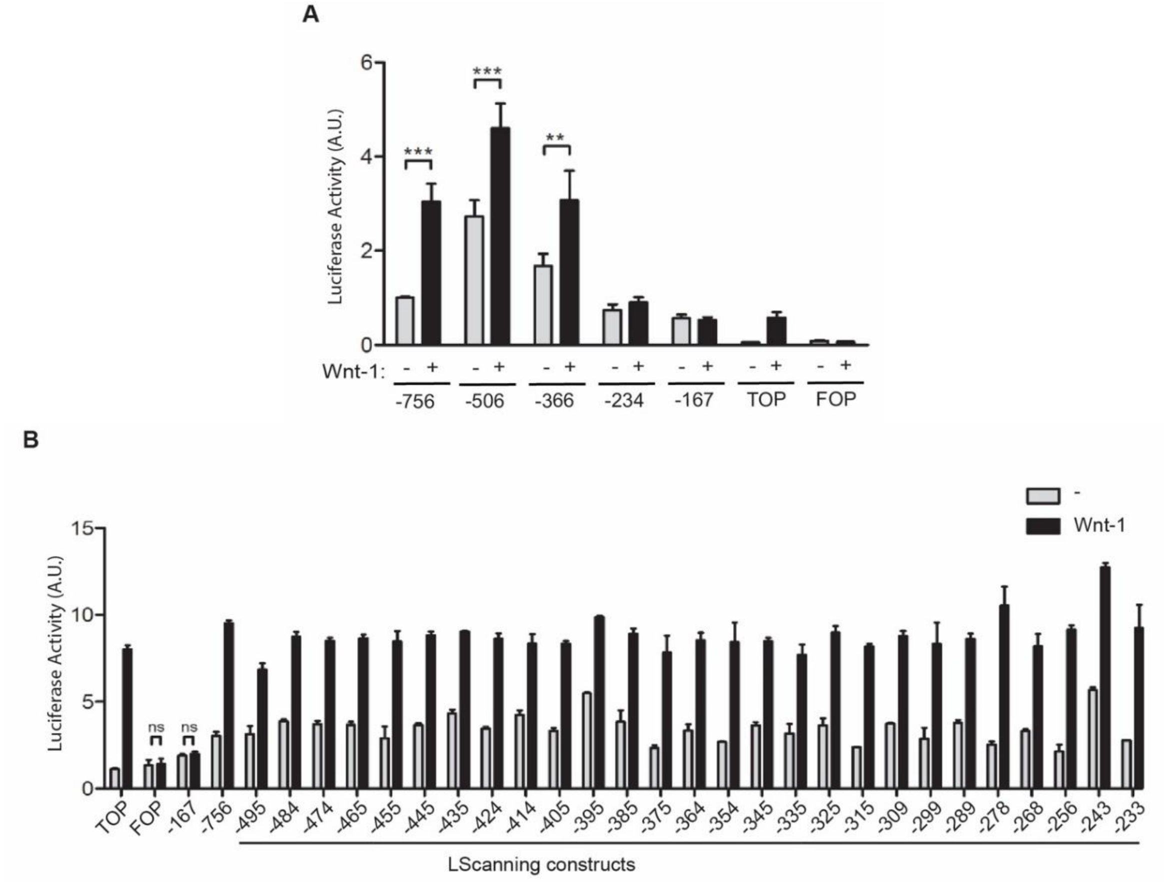

Figure S1. RhoU promoter analysis in MEF cells. (**A**) Cells were transiently transfected with the indicated RhoU promoter constructs or with the TOP-flash and FOP-flash positive and negative control plasmids (TOP, FOP). A SEAP-expressing vector was included as an internal control for transfection efficiency. Transfected cells were co-cultured for 24 h with wild type or WNT1-expressing HEK-293 cells. Luciferase activity was normalized to SEAP activity. Data are shown as mean ± SEM of independent experiments. ** $p < 0.01$, *** $p < 0.001$. (**B**) The indicated linker scanning (Lscanning) constructs based on the −756 RhoU-promoter fragment, along with the wild type −756 construct and the TOP and FOPflash controls were transiently transfected as above and tested for WNT1-responsiveness. Induction was always significant ($p < 0.001$), with the exception of the −167 and FOP negative controls (ns, non significant). A.U., arbitrary units. Data are shown as mean ± SEM of 4 independent experiments.

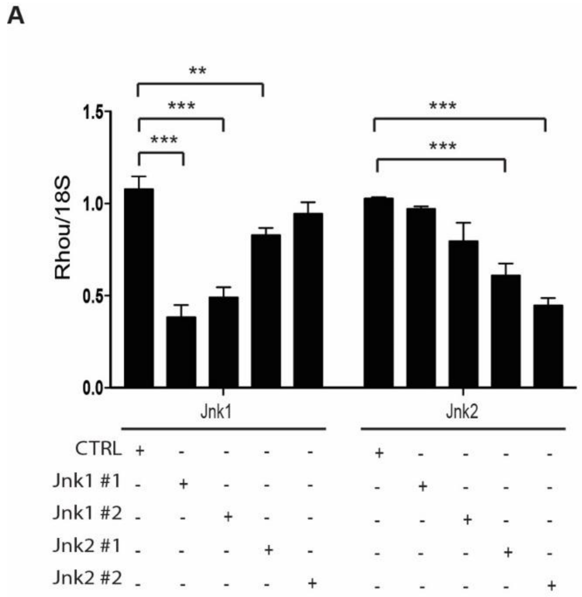

Figure S2. Downregulation of Jnk1 or Jnk2 mRNAs by the indicated shRNAs as measured by qRT-PCR in MEF cells. Data are shown as mean ± SEM of 3 independent experiments. ** $p < 0.01$, *** $p < 0.001$.

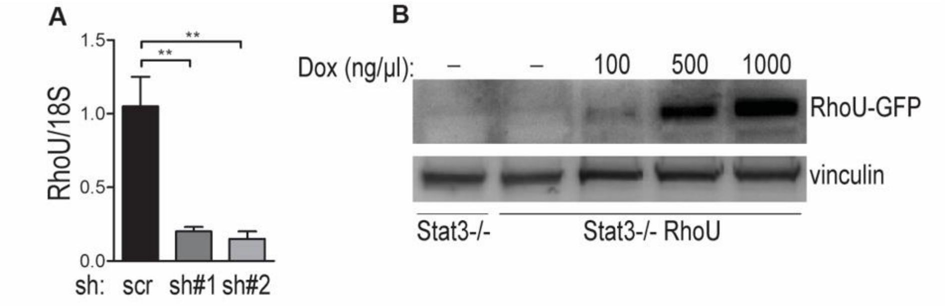

Figure S3. Manipulation of RhoU levels in MEF and MDA-MB-231 cells. (**A**) Lentiviral-mediated shRNA silencing of RhoU mRNA in MDA-MB-231 cells, measured by qRT-PCR upon infection with two distinct shRNA sequences (sh1, sh2) or with a control scrambled vector (scr). ** $p < 0.01$; $n = 4$. (**B**) RhoU-GFP expression levels in $Stat3^{-/-}$ MEF cells either untransfected or stably transfected with a RhoU-GFP TetON construct ($Stat3^{-/-}$ RhoU) and stimulated with the indicated doses of doxycycline (Dox), measured by Western blot with an anti-GFP monoclonal antibody.

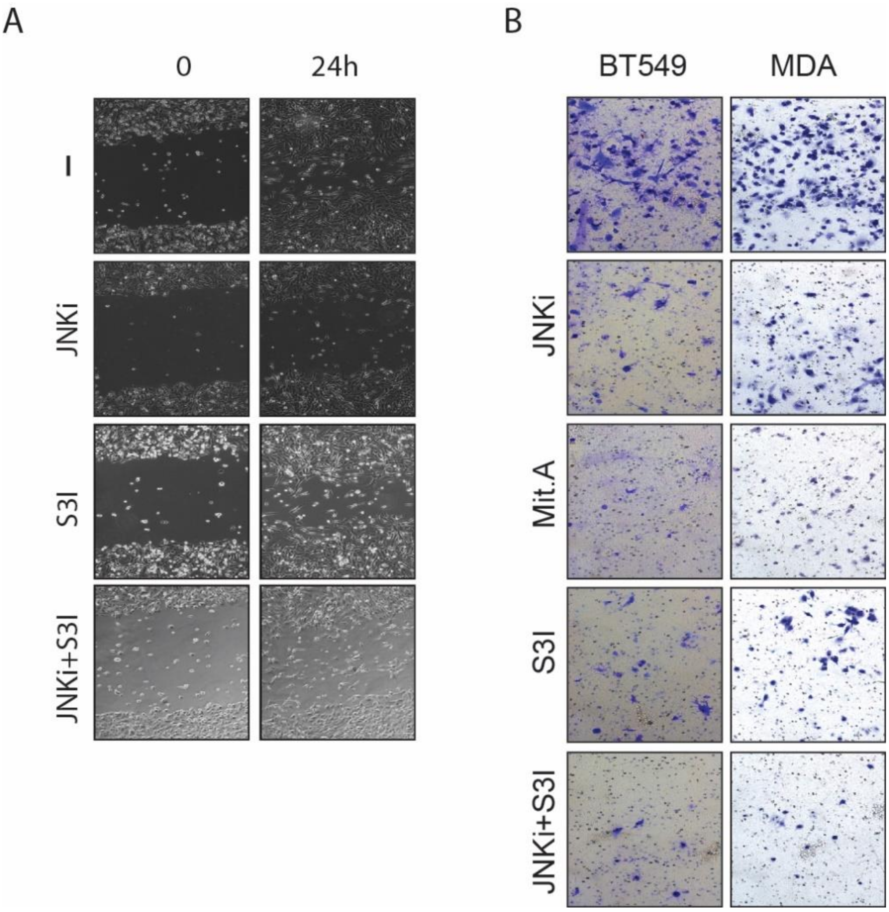

Figure S4. Migration and invasion assays upon treatment with various inhibitors. Representative pictures showing wound healing or transwell invasion assays with MDA-MB-231 (**A**,**B**) and BT-549 (**B**) cells treated either with the indicated inhibitors. Fields of View: 960 × 713 µm.

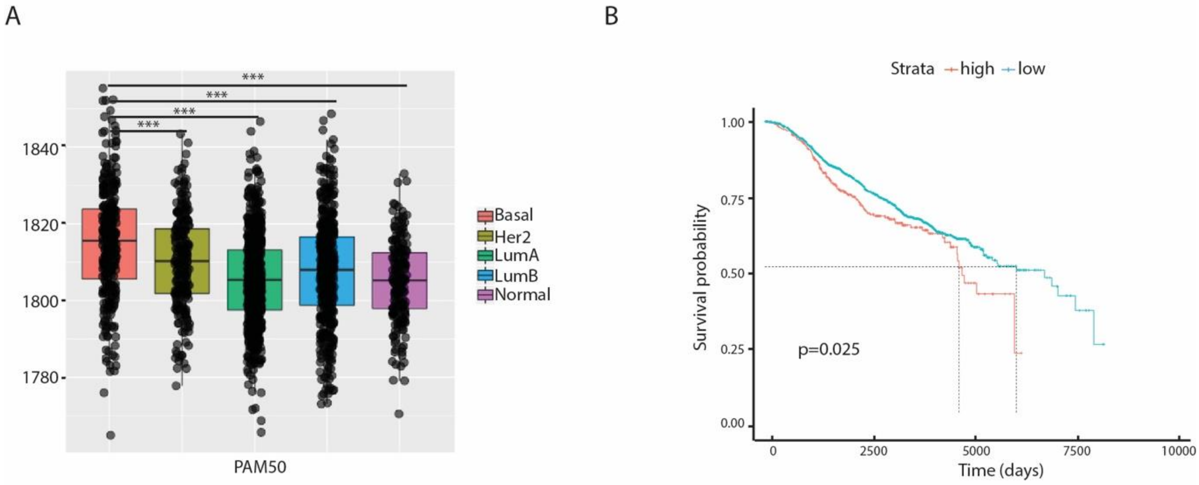

Figure S5. SP1 and STAT3 can functionally cooperate to enhance tumor aggressiveness independently of the tumor subtype. (**A**) Boxplot showing the distribution of the SP1-S3 score in the 5 breast cancer subtypes, significantly higher in basal-like tumors. * $p < 0.05$, ** $p < 0.01$, *** $p < 0.001$ between the indicated groups. (**B**) Kaplan-Meier plot of overall survival for breast tumor patients, as a function of time in days. Patient samples from the METABRIC database, excluding basal-like tumors, were subdivided according to low or high SP1-S3 score (median value). Even A high SP1-S3 score is predictive of poor prognosis.

Table S1. Total Binding Affinity of JASPAR Transcription Factors (TF) with the mouse RhoU promoter.

TF	rank
MZF1_5-13	0.995208285029755
ZNF354C	0.99408764201252031
ETS1	0.98334492619213232
MZF1_1-4	0.95969549424221345
Mycn	0.95749285107040727
Myc	0.95617899373985626
PPARG::RXRA	0.95204420743488682
Nr2e3	0.94292449184635596
Myf	0.94010356287193753
znf143	0.93426849060978434
INSM1	0.92453048921864134
EWSR1-FLI1	0.91869541695648815
ELK1	0.90644562949223273
SP1	0.89539377077053872
Arnt::Ahr	0.87727026818146692
Spz1	0.8758791251255893
RREB1	0.87421748203106886
ELF5	0.87309683901383417
PLAG1	0.86865290980755849
SPI1	0.85632583661797668
Esrrb	0.85168869309838469
BRCA1	0.83766133395161912
Sox17	0.83731354818764969
PBX1	0.83553597650513955
TLX1::NFIC	0.82943040420434344
Zfx	0.82417497488213931
REST	0.81621454517350644
ESR1	0.80983847283406751
RUNX1	0.80879511554215933
Egr1	0.80411932915990414
ESR2	0.80033232861890413
Klf4	0.79449725635675095
USF1	0.79306747043821002
GATA3	0.77548496792642396
Pax5	0.76779503825643403
NR3C1	0.76601746657392378
Pax4	0.76439446634206665
CTCF	0.76358296622613808
Gfi	0.7626941803848829
Sox2	0.75589303655614803
GABPA	0.75496560785222966
SPIB	0.74611639230234172
MYC::MAX	0.74244532034933153
Foxd3	0.73232089033155578
TFAP2A	0.71002395857485123
SOX9	0.70299095757013674
Stat3	0.69924259989179993
NHLH1	0.69456681350954474
Myb	0.68958188422598343
SRF	0.68757245536749367
Tal1::Gata1	0.68223974031996293
HNF4A	0.67640466805780974

HIF1A::ARNT	0.65801066543009501
E2F1	0.63888244841177833
Hand1::Tcfe2a	0.6313857330551047
IRF2	0.62976273282324757
RXR::RAR_DR5	0.62856480408068627
GATA2	0.62728958961279857
Zfp423	0.62543473220496171
NFATC2	0.61187108741015539
MAX	0.61183244454749208
FEV	0.5974959424994204
SRY	0.59479094211299177
NFKB1	0.58733286961898135
NFIC	0.58273436896205266
Arnt	0.58227065461009353
TAL1::TCF3	0.58049308292758328
TEAD1	0.57527629646804235
Nkx3-2	0.5626014375144911
REL	0.53759950537135792
ARID3A	0.5313393616199088
SOX10	0.5263930751990108
Lhx3	0.52055800293685761
NF-kappaB	0.51823943117706162
Hltf	0.51584357369193912
Nkx2-5	0.4831130690161527
Gata1	0.48017621145374451
Ddit3::Cebpa	0.47078599582657082
MIZF	0.44914599273514183
NR1H2::RXRA	0.44377463482494783
ELK4	0.44137877733982533
EBF1	0.44033542004791715
Pou5f1	0.43175670453667209
NFE2L1::MafG	0.42881984697426384
STAT1	0.42093670299095759
IRF1	0.41888863126980447
En1	0.41884998840714122
RELA	0.41684055954865135
Sox5	0.41363320194760028
TP53	0.41119870159981453
Mafb	0.40710255815750829
YY1	0.39280469897209985
RXRA::VDR	0.39210912744416104
Pax6	0.38774248396321198
Tcfcp2l1	0.38720148388592629
NR2F1	0.36532962361851767
NKX3-1	0.36053790864827268
PPARG	0.33855011979287425
NR4A2	0.32552747507535357
ZEB1	0.31265940180848595
HNF1B	0.28560939794419971
RORA_1	0.28460468351495477
Prrx2	0.27471211067315865
Pax2	0.26791096684442384
MEF2A	0.26586289512327071
T	0.25512017930288278
FOXC1	0.21535667362238195
HNF1A	0.21354045907720845

Evi1	0.18467424066774868
Foxq1	0.18254888322126903
FOXI1	0.17779581111368731
HOXA5	0.17327459618208516
FOXO3	0.15248473606924801
Nobox	0.15202102171728882
Pdx1	0.14823402117628873
CREB1	0.13181080454440064
NFYA	0.12400494628642089
FOXD1	0.12280701754385964
CEBPA	0.1195610170801453
FOXL1	0.096877656696808104
FOXF2	0.09571837081691012
Ar	0.091660870237267184
NFE2L2	0.091081227297318185
FOXA1	0.082966226138032312
HLF	0.079372439910348563
RORA_2	0.064842723548960507
TBP	0.052322436046062293
Foxa2	0.049385578483654068
NFIL3	0.046989720998531569
AP1	0.038449648349949767

The table shows a list of the 130 transcription factors described in the JASPAR Core Vertebrata database (downloaded November 2009), ranked according to their total binding affinity with the WNT-responsive region of the Rhou promoter (−756 to −235). A rank of 0.99 means that the affinity value with the Rhou promoter is higher compared to that shown by 99% of all gene promoters.

Table S2. See separated file.

Table S3. See separated file.

Table 4. Primers used to generate the RhoU promoter fusions.

Primer	Sequence
-1756 forward	5′-ACTAGGTACCGAATTCAGGTGGCCCAAGGGC-3′
-1316 forward	5′-GGCCGGTACCGCATCTCTGTACTTCAGGTTGTCC-3′
-756 forward	5′-GGCCGGTACCCGCTAACAAAGGGTGGTATCCTGC-3′
-506 forward	5′-GGCCGGTACCCCATGATCACCCTGCTTCTTCC-3′
-366 forward	5′-ACTAGGTACCCTGCCCTGCCCCCTCC-3′
-234 forward	5′-ACTAGGTACCGCTGGCTCCACCACG-3′
-167 forward	5′-GGCCGGTACCACAGCGCCCCCGTCTGC-3′
reverse	5′-TATCTCGAGGCCGCGAGACCAGCTGCC-3′

Table S5. Primers used to generate the Linker Scanning constructs.

Linker	Forward primer 5′-3′	Reverse primer 5′-3′
LSc -605	CCGAATTCAACACTCTCCCCCTCC	TTGAATTCGGTGCCTGCTTTTGCAG
LSc-595	CCGAATTCAACTCCAAACCAAGACAC	TTGAATTCGGCTGTGGGGCCTG
LSc -585	CCGAATTCAAAGACACTTGTGTGGAG	TTGAATTCGGGGGGAGAGTGCTG
LSc-575	CCGAATTCAAGTGGAGGAGCAAGG	TTGAATTCGGTGGTTTGGAGGGGG
LSc -565	CCGAATTCAAAAGGGCAGCTGGAGG	TTGAATTCGGACAAGTGTCTTGGTTTGG
LSc-555	CCGAATTCAAGGAGGAACAGGAAGAG	TTGAATTCGGGCTCCTCCACACAAG
LSc -545	CCGAATTCAAGAAGAGTGGAAGAAGC	TTGAATTCGGAGCTGCCCTTGC
LSc-535	CCGAATTCAAAGAAGCAGGGTGATCATGG	TTGAATTCGGCTGTTCCTCCAGCTGC
LSc -525	CCGAATTCAATGATCATGGTGAGAGG	TTGAATTCGGTCCACTCTTCCTGTTC

LSc-515	CCGAATTCAAGAGAGGGAGACCG	TTGAATTCGGCCCTGCTTCTTCCAC
LSc-505	CCGAATTCAACCGCGGAAAGGAG	TTGAATTCGGACCATGATCACCCTG
LSc -495	CCGAATTCAAGAGGTTGTGGAGTAG	TTGAATTCGGTCTCCCTCTCACC
LSc-484	CCGAATTCAAGTAGAAAGAGAATAGGGGG	TTGAATTCGGCCTTTCCGCGGTCTC
LSc -474	CCGAATTCAAAATAGGGGGCGACC	TTGAATTCGGTCCACAACCTCCTTTCC
LSc-465	CCGAATTCAACGACCTAAGGAGGG	TTGAATTCGGTCTTTCTACTCCACAACC
LSc -455	CCGAATTCAAAGGGAGAAGGAGGAG	TTGAATTCGGCCCCCTATTCTCTTTC
LSc-445	CCGAATTCAAAGGAGAGGGAAGAG	TTGAATTCGGCCTTAGGTCGCCC
LSc -435	CCGAATTCAAAGAGAAAGAGCCGGG	TTGAATTCGGCCTTCTCCCTCCTTAG
LSc-424	CCGAATTCAACGGGAGGGAAGG	TTGAATTCGGTTCCCTCTCCTCC
LSc -414	CCGAATTCAAGGGGGAACAGAAGG	TTGAATTCGGGCTCTTTCTCTTCCCTC
LSc-405	CCGAATTCAAGAAGGATGGGGAGG	TTGAATTCGGTCCCTCCCGGC
LSc -395	CCGAATTCAAGAGGAGGGGGC	TTGAATTCGGTGTTCCCCCTTCCC
LSc-385	CCGAATTCAACAGGGCAGGAGG	TTGAATTCGGCCCATCCTTCTGTTC
LSc -375	CCGAATTCAAGGAACTGGAGAGC	TTGAATTCGGCCCCCTCCTCC
LSc-364	CCGAATTCAAGCAAGAGGAGGAAAG	TTGAATTCGGCTCCTGCCCTGC
LSc -354	CCGAATTCAAGAAAGGAAGCTTGGG	TTGAATTCGGTCTCCAGTTCCTCC
LSc-345	CCGAATTCAACTTGGGAAGGAGCC	TTGAATTCGGTCCTCTTGCTCTCC
LSc -335	CCGAATTCAAAGCCGGGAAAGG	TTGAATTCGGCTTCCTTTCCTCCTC
LSc-325	CCGAATTCAAGGGGGGTGGG	TTGAATTCGGCCTTCCCAAGCTTCC
LSc -315	CCGAATTCAAGGGATGAGAAGCC	TTGAATTCGGTTTCCCGGCTCC
LSc-309	CCGAATTCAAAGAAGCCCACAGCAGG	TTGAATTCGGCCCCCCCTTTCCCG
LSc -299	CCGAATTCAAAGCAGGGGCCAG	TTGAATTCGGCATCCCCCCACC
LSc-289	CCGAATTCAAAGGGGTAGCTCGG	TTGAATTCGGGTGGGCTTCTCATCC
LSc -278	CCGAATTCAAGGGGTCCGCG	TTGAATTCGGTGGCCCCTGCTG
LSc-268	CCGAATTCAAGGGTTGGCGTGG	TTGAATTCGGGAGCTACCCCTGG
LSc-256	CCGAATTCAAGAGCCAGCTAGTCC	TTGAATTCGGCACGCGGACCCCG
LSc-242	CCGAATTCAAGTCCAGCCGCAC	TTGAATTCGGCACCACGCCAACC
LSc-233	CCGAATTCAAACCGTTTCGAGGCG	TTGAATTCGGTAGCTGGCTCCACC

Table S6. Primers used for qRT-PCR.

Gene	Forward primer 5′-3′	Reverse primer 5′-3′	Probe
Mus musculus RhoU	ACGGCCTTCGACAACTTCT	ACTCATCCTGTCCTGCAGTGT	78
Homo sapiens RhoU	GACTCCAACTCTGTGACACTGC	ATGAGGGGCTCACGACACT	4
Homo sapiens WNT5a	AGGGCTCCTACGAGAGTGCT	GACACCCCATGGCACTTG	/
Homo sapiens WNT5b	AGAAGAACTTTGCCAAAGGA	CTACGTCTGCCATCTTATACAC	/
Mus Musculus MAPK8 (JNK1)	AGAAACTGTTCCCCGATGTG	GGATAACAAATCTCTTGCCTGAC	33
Mus Musculus MAPK9 (JNK2)	GCTTGTTAAATGTGTTCACACC AC	TTCTCTCATGGTCCAGTTCCA	/
Homo sapiens Ror2	GTGACCTTTGTAGACTTTCC	ATTCTGTACATCTTGGTCCC	/
Homo sapiens Ror1	GATTAGAAACCTCGACACCAC	GCAAAGACTCCATAGACGG	/

© 2019 by the authors. Submitted for possible open access publication under the terms and conditions of the Creative Commons Attribution (CC BY) license (http://creativecommons.org/licenses/by/4.0/).
